# Structure–Activity Relationship and Molecular Docking of a Kunitz-Like Trypsin Inhibitor, Kunitzin-AH, from the Skin Secretion of *Amolops hainanensis*

**DOI:** 10.3390/pharmaceutics13070966

**Published:** 2021-06-26

**Authors:** Yuqing Chen, Xinping Xi, Chengbang Ma, Mei Zhou, Xiaoling Chen, Zhuming Ye, Lilin Ge, Qinan Wu, Tianbao Chen, Lei Wang, Hang Fai Kwok

**Affiliations:** 1 School of Pharmacy, Queen’s University Belfast, 97 Lisburn Road, Belfast BT9 7BL, UK; ychen52@qub.ac.uk (Y.C.); c.ma@qub.ac.uk (C.M.); m.zhou@qub.ac.uk (M.Z.); x.chen@qub.ac.uk (X.C.); zye04@qub.ac.uk (Z.Y.); t.chen@qub.ac.uk (T.C.); l.wang@qub.ac.uk (L.W.); 2 Institute of Translational Medicine, Faculty of Health Sciences, University of Macau, Avenida da Universidade, Taipa, Macau, China; 3 College of Pharmacy, Nanjing University of Chinese Medicine, Nanjing 210023, China; gelilin@njucm.edu.cn (L.G.); wuqn@njucm.edu.cn (Q.W.)

**Keywords:** Kunitz-like trypsin inhibitors, structure–activity relationship, molecular docking

## Abstract

Kunitz-like trypsin inhibitors are one of the most noteworthy research objects owing to their significance in pharmacological studies, including anticarcinogenic activity, obesity regulation and anticoagulation. In the current study, a novel Kunitz-like trypsin inhibitor, Kunitzin-AH, was isolated from the skin secretion of *Amolops hainanensis*. The novel peptide displayed a modest trypsin inhibitory activity with the inhibitor constant (*K_i_*) value of 1.18 ± 0.08 µM without inducing damage to healthy horse erythrocytes. Then, a series of shortened variants of Kunitzin-AH were designed by truncating a peptide loop and site mutation inside the loop to illustrate the structure–activity relationship of the trypsin inhibition function. Among the variants, a significant decrease was observed for the Cys-Cys loop domain, while the extension of an Arg at N-terminus (RCKAAFC) retained the inhibitory activity, indicating that the -RCK-motif is essential in forming the reactive domain for exerting the inhibitory activity. Furthermore, substitutions of Ala by hydrophobic or hydrophilic residues decreased the activity, indicating suitable steric hindrance provides convenience for the combination of trypsin. Additionally, the conformational simulation of the analogues processed with Chimera and Gromacs and further combination simulations between the peptides and trypsin conducted with HDOCK offered a potential opportunity for the natural trypsin inhibitory drug design. The truncated sequence, AH-798, may be a good replacement for the full-length peptide, and can be optimized via cyclization for further study.

## 1. Introduction

Kunitz-type trypsin inhibitors (K.T.I.s) are widespread in plants [1,2,3] and venoms [4,5], and the first Kunitz inhibitor was isolated from soybean in 1945 [6]. Since then, quantities of Kunitz-type inhibitors have been characterized from different species varying from plants to animals [7], and animal-derived K.T.I.s have obtained much focus in decades. Due to the wide distribution, in addition to inhibitory activity, K.T.I.s have been utilized in many other situations. These include potent anticarcinogenic activity in the animal-mimetic environment [8] through blocking urokinase-type plasminogen activator to suppresses ovarian cancer cell invasion [9], regulating obesity and metabolic disorders via cholecystokinin release, resulting in a reduction in hunger in the brain and promoting meal cessation [10], and hampering blood clotting by hindering blood coagulation enzymes [11].

Generally, inhibitors in the Kunitz-type family have some structural features in common. On the one hand, Kunitz inhibitor is a single or double polypeptide chain with the molecular weight ranging from 18 kDa to 24 kDa, and it usually contains a β-trefoil fold, four residues of cysteine forming two disulfide bonds which play an essential role in stabilizing its structure. On the other hand, unlike the Bowman–Birk family, K.T.I.s have only one reactive site in enzyme inhibitory reactions [12], and they can combine with trypsin reversibly in a substrate-like manner, giving rise to a Michaelis complex, and then shape a stabilized acyl-enzyme complex, which will not be influenced by the hyper-slow hydrolysis reaction aiming at inhibitors. Specifically, the classic mechanism between K.T.I.s and trypsin involves a tight, non-covalent competitive interaction, in which K.T.I.s will directly block the trypsin active site (Ser195), and then an anti-parallel β-sheet between enzyme and inhibitor will be formed. Therein, the inhibitory reaction appears to be stable, and there is no space for the enzyme to approach other proteins to break down [13,14].

Animal origin Kunitz-type inhibitors provide useful approaches for the study of structure–activity relationships between protease and their inhibitors, such as Bi-KTI derived from bumblebee venom [15], Pr-mulgins found in snake venom [16], and Kunizins isolated from the skin secretion of ranid frogs [17].

**Figure 1:**
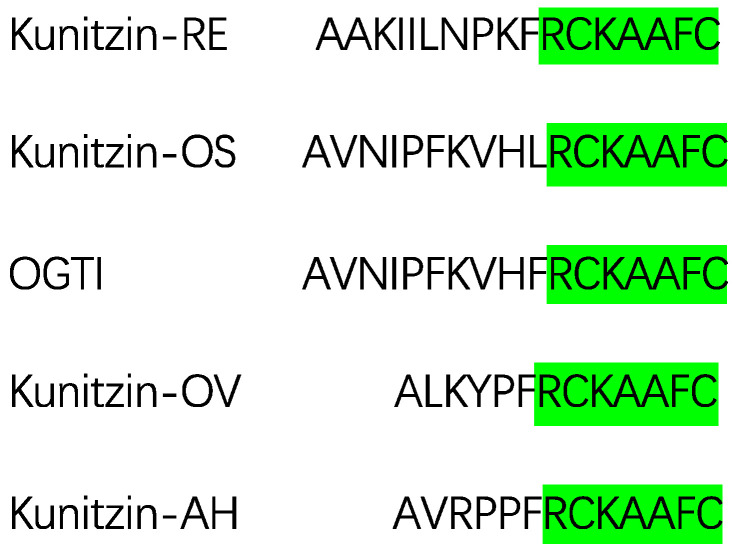
The mature peptide sequence of Kunitzin-AH from the skin secretion of *Amolops hainanensis* compared with previously identified kunitzins. Fully conserved domains were marked in green.

Similar sequences were identified from frog skin secretions as shown in Figure 1, namely, Kunitzin-RE, Kunitzin-OS [17], OGTI [18] and Kunitzin-OV [19]. In this study, we found a novel Kunitz-like trypsin inhibitor from the skin secretion of Hainan cascade-frog *Amolops Hainanensis*, named Kunitzin-AH. These inhibitors share a fully conserved heptapeptide region -RCKAAFC-, which is marked with green in the figure. In previous studies, it has been shown that the Arg residue adjacent to the Cys-Cys loop is a primary trypsin cleavage site. Moreover, the substitution of the Lys residue with Phe in the P1 position of the inhibitor led to the removal of inhibitory activity towards trypsin. Simultaneously, a significant decrease in the activity was also observed for the Cys-Cys domain [17,19]. These results indicated that the -RCK- motif was essential for forming the reactive domain for exerting the inhibitory activity. Therefore, in the current study, AH-798 (RCKAAFC) was designed, and interestingly, the extension of an Arg at the N-terminus retained the inhibitory activity. This finding inspired us to transfer the focus onto the structure–activity relationship of the loop region, which has not been investigated so far. Although quantities of articles focusing on Kunitz-like trypsin inhibitors have been published [7,11,12,14,17,18,19], research on the structure–activity relationship of their fully conserved loop domain is fairly limited. Further investigations on the novel peptide are trying to explain its structure–activity relationship, and the vital mission of the present study is to associate the alterations of the primary structures of the inhibitors with the changes in inhibition expression by use of modeling and molecular docking methods.

Here, a series of variants of shortened Kunitzin-AH was designed to evaluate the effects of hydrophobicity, aromaticity, integrity of disulfide bond, length, size and steric hindrance of the peptide and several variants were chosen to simulate the process of combination between inhibitors and the enzyme. Thus, it could represent a good starting point for enabling the protein designers to achieve the goal of specifically targeting these tiny proteins.

## 2. Results

### 2.1. Identification and Characterization of Kunitzin-AH from the Frog Skin Secretion

The full length of the novel peptide Kunitzin-AH precursor-encoding cDNAs was cloned from the skin secretion of *Amolops hainanensis*. Nucleotide and translated open-reading frame amino acid sequence of cloned cDNAs are exhibited in Supplementary Materials Figure S1. The translated open-reading frame was 66 amino acids in length and could be divided into separate parts as follows. To begin with, the putative signal peptide was encoded by the first 22 amino acid residues, which were marked with double underlining. The next 29 amino acid residues (mostly acidic residues) formed a ‘spacer’ region which was left blank and followed by a representative cleavage site -Lys-Arg- (-K-R-). The mature peptide (AVRPPFRCKAAFC) was marked with single-peptides, and there was an apparent ‘Rana box’ between Cya8 and Cys13 at the C-terminal of the peptide. The fractions of skin secretion sample from reverse-phase high-performance liquid chromatography (RP-HPLC; Figure S2a), which contained a peptide of identical molecular mass to that of the predicted mature peptide, were further analyzed by MS/MS fragmentation sequencing (Figure S2b,c), which validated the primary structure of Kunitzin-AH. As analyzed via basic local alignment search tool (BLAST) search (Figure 1), the hexapeptide loop region of the novel sequence displayed a high consistency with the previously discovered kunitzin peptides, namely, Kunitzin-RE, Kunitzin-OS, OGTI and Kunitzin-OV [17,18,19]. The fully conserved loop regions of the novel and previously identified sequences were highlighted in light green. Therefore, the novel peptide was assumed as a member of the Kunitz-like trypsin inhibitor peptide family—the kunitzins.

### 2.2. Motif-Targeted Peptide Design

A series of variants were designed and synthesized based on the primary structure of the fully conserved domain of Kunitzin-AH (Table 1). Sequences and physicochemical properties of Kunitzin-AH and its derivatives were shown in Table S1.

On the one hand, disulfide bonds are of great importance in stabling specific structures and maintaining bioactivities for proteins [20,21]. When those bonds are broken and reduced to sulfhydryl groups, structures of proteins will undoubtedly be rebuilt, and their biological activities will change more or less. The majority of trypsin inhibitors (including Kunitz-like, Bowman–Birk family and Kazal-like) have cysteines in their structures, and some of them can form intracellular disulfide bonds [12,22,23], which are considered as the main character in the interaction of inhibitors and enzymes. However, those inhibitors which lack the construction of disulfide bonds such as the insecticidal BrTI might have a function of inhibition as well [7]. Therefore, to determine whether the Cys-Cys loop domain was the active center of Kunitzin-AH, AH-1363, a modified sequence removing the cysteine at the C-terminal was designed. Meanwhile, to estimate the effect of the oxidation of the cysteine loop, an original sequence of Kunitzin-AH was synthesized, avoiding the process of oxidation, leaving the two sulfur atoms to exist as free sulfhydryl groups.

In addition, as Figure 1 showed, there were three distinguished amino acid residues outside the loop domain between Kunitzin-OV and Kunitzin-AH; they displayed similar trypsin inhibitory activity with the *K_i_* values of 3.04 µM and 1.18 µM, respectively. Simultaneously, the removal of the N-terminal domain did not affect the inhibitory activity, while the changes in the amino acid constitution caused the outstanding influence of the inhibitory potency, indicating the N-terminus elements of the inhibitors play a less important role in the interaction with trypsin. Various studies have suggested that the length and size of the peptide [24], hydrophobicity [25] and aromaticity [18] could affect the trypsin inhibitory activity; subsequently, AH-1447 has been designed with a Lys substituting Phe at C-terminus. Following this, Trp and Tyr, both with their own aromatic ring in their structures, have been applied to alter the Phe at the same position (AH-837 and AH-814) to adjust the hydrophobicity and aromaticity of the peptide. A previous study in our lab showed that a significant decrease was observed for the Cys-Cys domain [17], considering the Arg residue might be the active site in the interaction with the enzyme; in our current research, AH-798 (RCKAAFC), the extension of an Arg at N-terminus, has been designed. Furthermore, substitutions of Ala by hydrophobic (Ile, Leu and Gly) or hydrophilic (Asp and Gln) residues were both applied to alter the steric hindrance (AH-880-I, AH-880-L, AH-770, AH-884 and AH-912).

### 2.3. Trypsin Inhibition and Hemolysis Assays

Kunitzin-AH and its analogues were subjected to protease inhibition assay against trypsin and hemolysis assays towards horse erythrocytes. Consequently, Kunitzin-AH functioned as a modest trypsin inhibitor with the *K_i_* value of 1.18 ± 0.08 µM, which exhibited similar inhibitory potency with previously identified kunitzins, Kunitzin-RE, Kunitzin-OS, OGTI and Kunitzin-OV, with the *K_i_* values of 5.56, 7.56, 0.40 and 3.04 µM, respectively [17,18,19]. All of the synthetic peptides displayed trypsin inhibitory activity with different levels of potency (Table 1) and no hemolytic activity towards normal horse erythrocytes. By extension, the analogues with a disrupted disulfide bridge, AH-1363 and AH-unoxidized, displayed eighty-fold and fifty-fold larger *K_i_* values than those of the original sequence, which could be deemed to lose the activity of inhibition. Moreover, the substitution of Phe with a polar residue (Lys) at the C-terminus largely decreased the inhibitory potency, which could be observed through the result of AH-1447, while the Tyr-substituted sequence, AH-814, displayed a more apparent decrease in activity than that of the Trp-substituted sequence, AH-837. Furthermore, the Ala-substituted derivatives, AH-880-I, AH-880-L, AH-770, AH-884, and AH-912, also decreased the inhibitory activity. Among the analogues, AH-798, the extension of an Arg at N-terminus, was the only one that retained the similar *K_i_* value with Kunitzin-AH.

### 2.4. Modeling and Molecular Docking Simulation

#### 2.4.1. Molecular Dynamics (MD) Simulation

**Figure 2:**
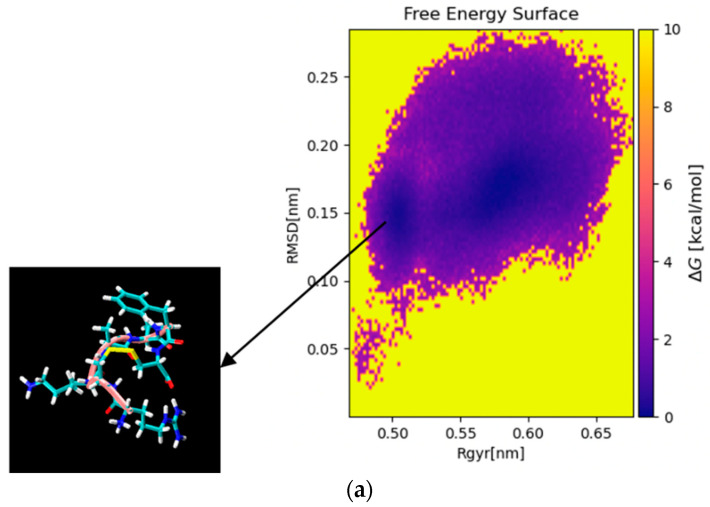

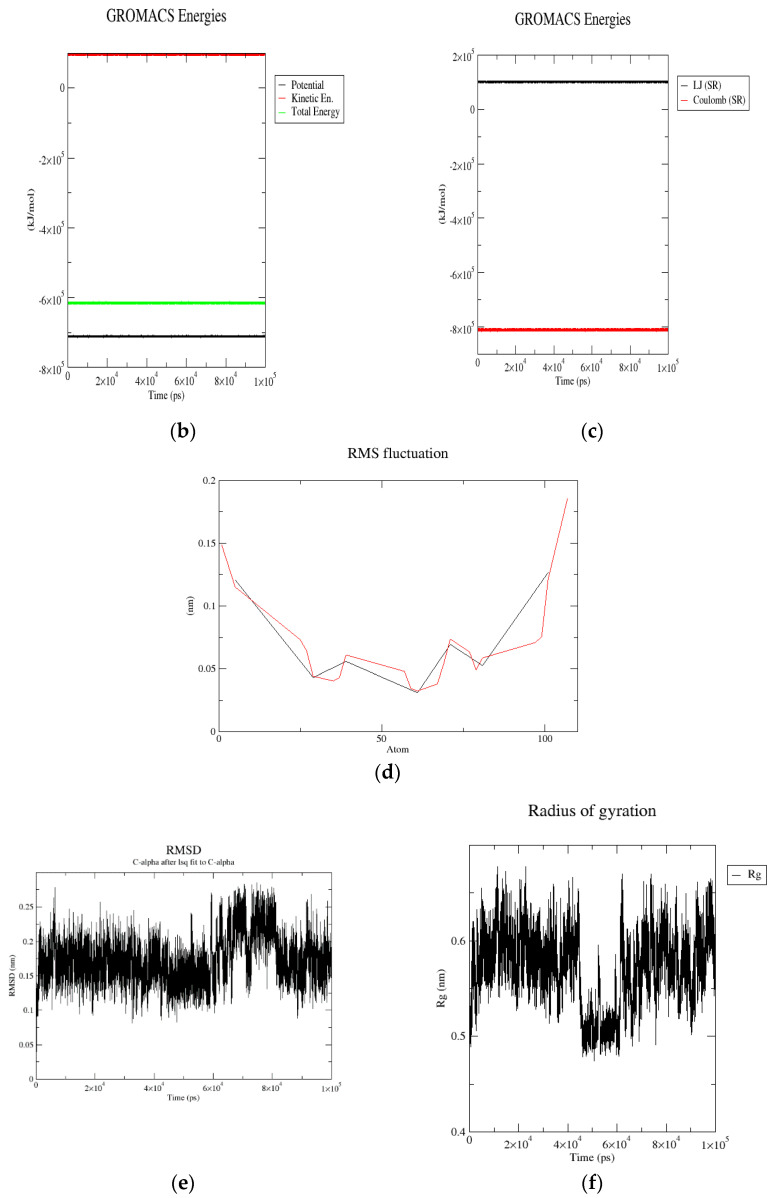
(**a**) Free energy surfaces (in kcal/mol) for the aggregation of AH-798 forming a disulfide bridge in terms of the radius of gyration (Rgyr) and the root mean square deviation (RMSD). The darker region represents the lower energy of the structure, and the conformation with the lowest energy is indicated with an arrow. (**b**) Potential energy (black), kinetic energy (red) and total energy (green) fluctuation of the system during the production molecular dynamics simulation within 100 ns; (**c**) Lennard-Jones Short Range potential (black) and Coulomb short-range force (red) fluctuation of the system during the production molecular dynamics simulation within 100 ns. (**d**) Root mean square fluctuation (RMSF) of the Cα in the structure of AH-798 (indicated with black lines) and the carbon backbone of the structure (indicated with red lines). (**e**) The root mean square deviation (RMSD) values of the Cα in the structure of AH-798 during the production molecular dynamics simulation within 100 ns. (**f**) Radius of gyration (Rg) of the whole conformation during the molecular dynamics simulation within 100 ns.

To display the aggregation patterns of the selected short peptides, molecular dynamic simulations were applied in this section. Specifically, MD simulations were conducted on AH-798 to evaluate the energy and stability of the constructed structure. As shown in Figure 2, the lowest energy structure was selected based on the free energy surface calculation in terms of the radius of gyration (Rgyr) and the root mean square deviation (RMSD). The whole conformation displayed stability with neglectable fluctuation during the production of molecular dynamics simulation within 100 ns (Figure 2b,c). Additionally, root mean square fluctuation (RMSF) of the Cα and the carbon backbone of the structure exhibited similar fluctuation between 0.025 nm and 0.2 nm (Figure 2d). Furthermore, the root mean square deviation (RMSD) values of the Cα in the structure fluctuated between 0.05 nm and 0.3 nm, while the radius of gyration (Rg) of the whole conformation fluctuated between 0.45 nm and 0.7 nm (Figure 2e,f). These results indicated that the selected structure of AH-798 was a suitable input for molecular docking simulation. The models of the other two analogues, AH-837 and AH-884, were both modified from the template model of AH-798, and the lowest-energy structures were selected through the identical process (Figures S4 and S5).

#### 2.4.2. Ligand–Protein Docking Simulations with HDOCK

**Figure 3:**
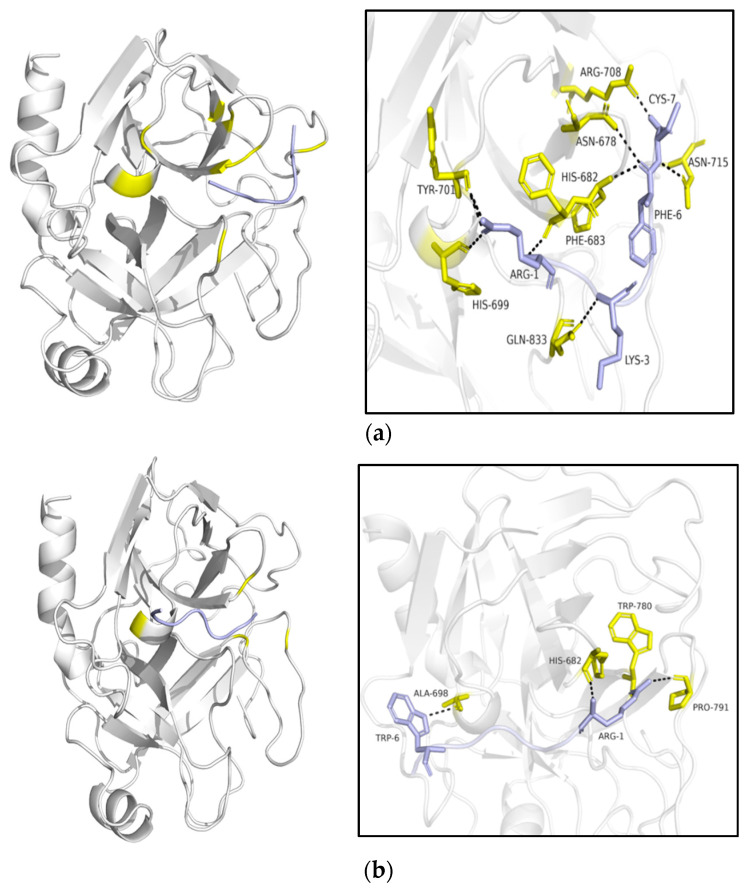

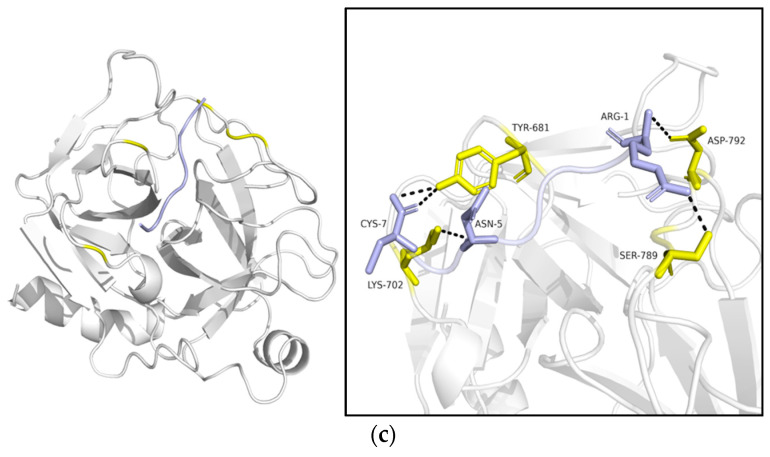
Visualization of the mimetic molecular docking of the combination between the inhibitors and trypsin (bovine pancreas proteasome, PDB ID: 1S0Q) by HDOCK. Ligands were highlighted in blue, and the receptor was represented in grey. (**a**) interaction between AH-798 and trypsin; (**b**) interaction between AH-837 and trypsin; (**c**) interaction between AH-884 and trypsin. Residues involved in the combination are marked with yellow (receptor) and blue (ligand), and the hydrogen bonds are expressed by black dashed lines among the residues.

HDOCK webserver was applied here as a hybrid docking strategy to implement inhibitors–trypsin docking at the molecular level. The shape-based pairwise scoring function allows it to search all the translation and rotation spaces and then rank the top translations with the best shape complementarities among thousands of situations. PyMOL was employed to realize the visualization of the combination. The highest-ranked complexes are displayed in Figure 3. Three sequences with significantly different levels of potency of inhibitory activity were submitted to the program, namely, AH-798, AH-837 and AH-884. It was indicated through the docking results that the Phe 683, His 699, Tyr 701; Gln 833; His 682, Asn 678, Asn 715, and Arg 708 residues of the trypsin (receptor) could bind with Arg 1; Lys 3; Phe 6 and Cys 7 residues in AH-798 (Ligand), respectively. Moreover, AH-837, His 682, Try 780, and Pro 791 of trypsin could interact with Arg 1, and the Ala 698 was able to combine with Trp 6 of the receptor. In addition, for AH-884, the position of the combination was observed to display an adjustment. Ser 789, Asp 792, Lys 702, and Tyr 681 residues of the trypsin were considered to attach to Arg 1, Asn 5 and Cys 7 residues of the AH-884 ligand.

These simulated figures provided evidence of the interaction between inhibitors and the bovine pancreas proteasome, and the highest docking score between AH-798 and the receptor might explain the reason why AH-798 possessed the most potent inhibitory activity among these three sequences (Table 2).

## 3. Discussion

Kunitz-like trypsin inhibitors (K.T.I.s) are one of the most noteworthy research objects owing to their significance in pharmacological studies. In the giant family of identified K.T.I.s, animal-derived inhibitors exhibit great research significance in their structure–activity relationship [15,16,17]. Here, we found a novel Kunitz-like trypsin inhibitor peptide from the skin secretion of *Amolops hainanensis*, named Kunitzin-AH. It possesses the fully conserved hexapeptide loop region ‘-RCKAAFC’ with previously identified kunitzins [17,18,19] and undisputedly displays modest trypsin inhibitory activity with the *K_i_* value of 1.18 µM.

In the current study, a series of variants of shortened Kunitzin-AH was designed, and all the synthesized analogues of Kunitzin-AH exhibited decreased inhibitory activity more or less, except for AH-798 (*p* < 0.05). It could be indicated that for Kunitzin-AH, formation of the disulfide bond, length and size of the peptide, hydrophobicity and aromaticity affected the trypsin inhibitory activity. As the previous study showed, a significant decrease was observed for the Cys-Cys loop domain [17]. Additionally, the formation of the disulfate bridge affected the structure when binding to trypsin [12]. However, the extension of an Arg at the N-terminus retained the inhibitory activity, indicating the -RCK-motif is essential for forming the reactive domain for exerting the inhibitory activity. Furthermore, substitutions of Ala by hydrophobic (Ile, Leu and Gly) or hydrophilic (Asp and Gln) residues both decreased the activity (*p* < 0.05), indicating suitable steric hindrance provides convenience for the combination of trypsin.

**Figure 4:**
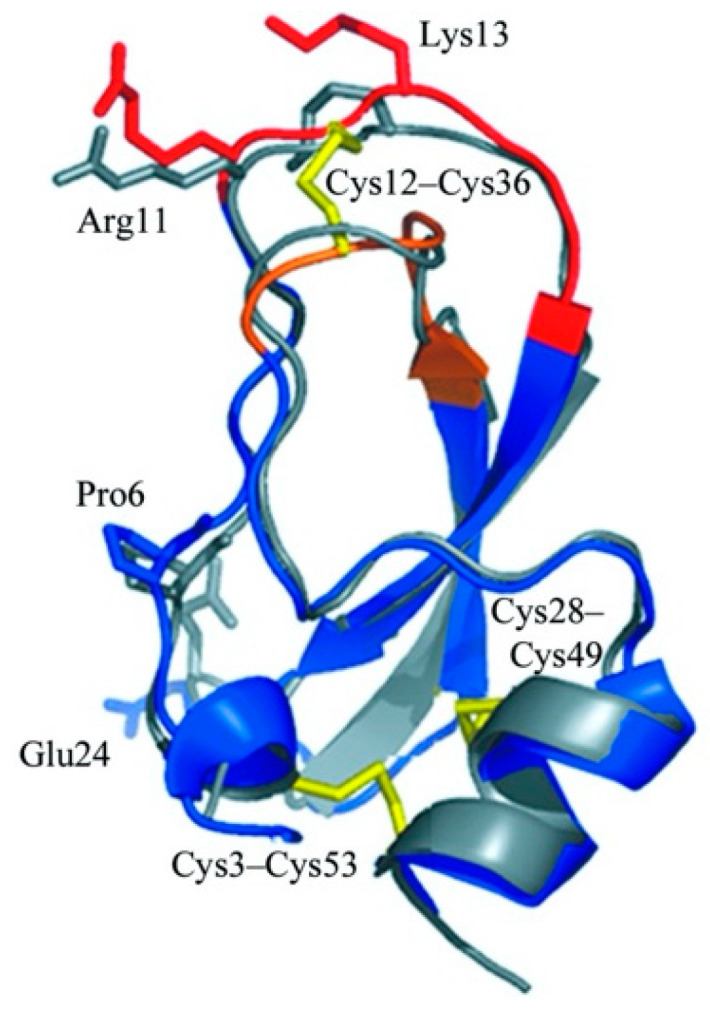
Structure of a classic Kunitz-like trypsin inhibitor, rShPI-1A. The figure was adjusted from the RCSB protein data bank [28], CC BY 4.0 license. PDB ID: 3OFW. The disulfide bonds were represented in yellow, and the binding loops were marked in red and brown.

According to the top-ranked docking models of three variants with apparently discrepant inhibitory potency, several reasons can be suggested that might contribute to the strongest activity of AH-798 among the variants. Firstly, among the three models, Arg 1 was a fundamental requirement in the interaction, which further illustrated the distinct inhibitory difference between AH-798 and the bold Cys-Cys loop [17]. Moreover, although both AH-798 and AH-837 could bind with the trypsin from the same position, much fewer residues in AH-837 participated in the interaction than those in AH-798, and thus fewer hydrogen bonds were formed simultaneously. In the process of inhibition, the formation of hydrogen bonds is responsible for the stabilization of the complexes, which consist of the cleavage residues of the inhibitors and the active ‘pocket’ of the trypsin [14]. Therefore, an advantage in the quantity of hydrogen bonds leads to the better inhibitory activity of AH-798. In addition, the Trp 6 residue resulted in the primary structure transformation of the inhibitor, hindering AH-837 to fold and form an angle such as AH-798, which might increase the steric hindrance of the inhibitor and thus cause inconvenience for inserting and weakening its inhibitory activity. Additionally, considering the structure of a classic trypsin inhibitor (Figure 4) [28], the -Arg 11 Cys 12 Lys 13 Gly14- motif, AH-884 (RCKNNFC) was designed and submitted to the docking tools as well, neither the actual experiments nor the docking results achieved expectations. In the highest-ranked simulated model, AH-884 is attached to a different position outside of the active ‘pocket’ of the trypsin. The active ‘pocket’ of the trypsin is known to be a specific site that undertakes the duty of cleavage in the interaction with either inhibitors or its substrate [12]. The altered combination sites of AH-884 made no difference in the proximity between trypsin and the substrate, and consequently, AH-884 displayed much lower inhibition activity.

Compared to the full-length sequence, the truncated version, AH-798, displayed both pros and cons. On the one hand, AH-798 retained the inhibitory potency with a shorter length and a smaller loop than truncated Bowman–Birk inhibitors [29], acting as a good replacement for the full-length version. Nevertheless, no improvement in the inhibition was obtained, which might arise from degradation of the peptide in the physical environment. In view of this, a cyclization loop (Phe12-Arg2) has been introduced in sunflower trypsin inhibitor-1 (SFTI-1) to form a rigid structure [30], which could be employed to optimize the activity of AH-798 for further study.

To sum up, through the research on the structure–function activity, we found that the fully conserved loop domain of kunitzins (RCKAAFC) was the most significant factor in the interaction, and each element inside the loop was irreplaceable. As natural drug discovery is quite a hot topic nowadays, studies on the structure–activity relationship of the novel skin-derived trypsin inhibitory peptide, Kunitzin-AH, might provide a potential opportunity for the drug design. Furthermore, the truncation of the full-length sequence of Kunitzin-AH could reduce the cost to a great extent in the aspect of synthesis and further manufacture, and a shorter sequence might possess more rapid clearance and lower accumulation in tissues. Additionally, the current research might provide a potential opportunity for the protein designers to achieve the expectations of specific targeting of the tiny proteins.

## 4. Materials and Methods

### 4.1. The Acquisition of Skin Secretion from Amplops Hainanensis

The frogs *Amolops hainanensis* were all mature adults and captured in the field in China. The secretion acquisition procedure was performed as described previously [31]. After being frozen in liquid nitrogen and lyophilized in an Alpha 1-2/LD freeze dryer (Martinchrist, Osterode, German), the sample was stored at −20 °C prior to use. This study was approved by the Nanjing University of Chinese Medicine Ethical Review Board—Approval Code: SYXK (S.U.) 2018-0048, 26 October 2018.

### 4.2. Molecular Cloning of cDNA Library from Amolops Hainanensis Skin Secretion

Five milligrams of lyophilized skin secretion were dissolved, and Polyadenylated mRNA was isolated from the skin secretion of *Amolops hainanensis* by use of a Dynabeads mRNA Direct Kit (Biotech, Southampton, UK). The first-strand cDNAs were obtained, and a cDNA library was then built according to the extracted mRNAs by a reverse transcription-polymerase chain reaction. Next, a B.D. Smart RACE cDNA Amplification Kit (B.D. Bioscience Clontech, Oxford, UK) was applied. The degenerate primer (DV-1; 5′-TAYGARATHGAYAAYMGICC-3′) (Y = C + T, R = A + G, M = A + C) was designed based on the highly conserved domain of the cDNAs from *Amolops hainanensis*.

### 4.3. Isolation and Identification of the Novel Peptide

An amount of 5 mg of lyophilized skin secretions was dissolved in 1 mL deionized water containing 0.05% (*v*/*v*) trifluoroacetic acid (TFA) and centrifuged at 5000× *g* for 20 min. The supernatant was taken out and shaken well, and the product was separated by reverse-phase high-performance liquid chromatography (RP-HPLC; Amersham Biosciences, Piscatawa, NJ, USA). The entire separation process lasted 240 min, and the fractions were automatically collected at 1-minute intervals based on the U.V. absorbance at 214 and 280 nm. Then, the different fraction samples were analyzed by matrix-assisted laser-desorption/ionization time-of-flight mass spectrometry (MALDI-TOF MS; Perspective Biosystems, Framingham, MA, USA), and the calculated peptides with the same molecular weight were analyzed using the LCQ-Fleet electrospray ion trap mass spectrometer (Thermo Fisher Scientific, San Jose, CA, USA) to perform MS/MS fragment analysis to infer their primary structures.

### 4.4. Solid-Phase Peptide Synthesis

Kunitzin-AH and its analogues were all synthesized from the C-terminus to the N-terminus applying standard Fmoc amino acids in a 2-channel Tribute automated peptide synthesizer (Protein Technologies, Tucson, AZ, USA) as previously described [32]. A final concentration of 0.01% hydrogen peroxide (Sigma-Aldrich, Dorset, UK) was added to the peptide solution with gentle stirring in order to form a disulfide bond.

### 4.5. Trypsin Inhibition Assays

The kinetic enzyme assay was performed as described in the previous study [17]. Substrate Phe-Pro-Arg-AMC was employed for investigating the hydrolysis of bovine trypsin. Trypsin working solution (10 µL trypsin stock solution (0.1 µM) + 9.99 mL HCl (1 mM)) and trypsin substrate working solution (50 µL substrate stock solution (Phe-Pro-Arg-AMC) (10 mM) + 9.95 mL PBS) were prepared prior to use. Trypsin inhibition assay contained three groups, namely positive control (20 µL PBS, 180 µL substrate working solution, 10 µL trypsin working solution), blank control (20 µL PBS + 10 µL HCl (1 mM) + 180 µL substrate working solution) and peptide group (20 µL peptide solution with the concentration ranging from 10 to 1000 µM, 180 µL substrate working solution, 10 µL trypsin working solution). The fluorescent strength released from hydrolysis of the substrate was monitored at 37 °C at the wavelength of 460 nm (excitation at 360 nm) in a POLARstar Omega Microplate Reader (B.M.G. Labtech, Offenburg, German). Each concentration of the sample was carried out in triplicate and repeated at least three times.

### 4.6. Hemolytic Activity Assay

Hemolysis assay was conducted through the incubation of peptides and 2% horse erythrocytes suspension (T.C.S. Biosciences Ltd. Buckingham, UK) at 37 °C for two hours as detailed in the previous study [19]. Afterwards, the positive control (considered as 100% hemolysis of cells) was conducted with 1% Triton X-100 (Sigma, Dorset, UK), while the negative control (0%) was treated with phosphate-buffered saline (PBS). The absorbance values were subsequently measured with a Synergy H.T. plate reader (BioTech, Minneapolis, MN, USA) and calculated by using Gen 5 software at a wavelength of 550 nm.

### 4.7. Molecular Dynamics Simulation and Ligand–Protein Docking

Several typical variants were chosen to simulate the process of combination between inhibitors and the enzyme. The probable three-dimensional structures of Kunitzin-AH and its variants were simulated and built by an open-source and universal molecular graphics visualization software, Chimera [33]. The obtained models were then subjected to Gromacs, an efficient program for molecular dynamics simulation [34]. The three-dimensional structure of bovine pancreatic trypsin (PDB ID: 1S0Q) was obtained from the RCSB protein data bank (https://www.rcsb.org/, accessed on 22 December 2020).

Several steps were included for the establishment of three-dimensional models of Kunitzin-AH and its variants. At first, the structure of the heptapeptide AH-798 (RCKAAFC) was created with UCSF Chimera software, including assigning bond orders, adding hydrogens, creating disulfide bridges and optimizing the structures. A cubic water box with sides of 7.5 nm was added to the system, and 150 mM sodium chloride (NaCl) was applied to model the typical physiological environment and to guarantee the electrically neutral condition of the entire system. The permitted maximum energy in the system was set as 500 kJ/mol/nm for energy minimization. After a topology file was produced, the obtained three-dimensional structures were equilibrated in Gromacs, and the a99SBdisp force field and TIP4P-D water model were applied to describe the intrinsically disordered proteins and water molecules, respectively. The system was subjected to a total of 100 ns of molecular equilibration dynamics, with an integration time of 2 fs. A Langevin dynamics approach was conducted to retain the temperature of the system at 298 K [35,36,37]. The structures of AH-837 and AH-884 were modified based on that of AH-798.

The peptide–protein docking system was applied in this strategy [38], and we found suitable inputs of three-dimensional structures of AH-798, AH-837 and AH-884 with the lowest energy for molecular docking simulation. The structures of peptides and trypsin were then submitted to the HDOCK server (http://hdock.phys.hust.edu.cn/, accessed on 6 January 2021) as ligand and receptor, respectively. The HDOCK server was applied as an FFT-based hybrid docking strategy to implement protein–protein docking at the molecular level. It owns a shape-based pairwise scoring function, which can search all the translation and rotation spaces and then rank the top translations with the best shape complementarities among thousands of situations. The highest-ranked HDOCK pose was selected and visualized by an open-source and popular biomolecular visualization software PyMOL [39].

The calculation of binding free energies (ΔGbind = Gcomplex − Gprotein − Gligand) was aimed at evaluating the affinities between inhibitors (ligand) and target enzyme (receptor) [40,41]. In HDOCK tools, it was represented with docking scores, and the larger scores meant a tighter affinity [27,42].

### 4.8. Statistical Analysis

Obtained data were analyzed to obtain the mean and standard error of the mean. All the statistical analysis was performed by GraphPad Prism 6 software (GraphPad, SanDiego, CA, USA).

## 5. Conclusions

In conclusion, Kunitzin-AH, a novel frog skin-derived bioactive peptide, is one of the smallest serine protease inhibitors ever found [18]. It is classified as a member of kunitzins and possesses potent trypsin inhibitory activity and fairly low toxicity simultaneously. Through the research on the structure–function activity, we found that the fully conserved loop domain of kunitzins (RCKAAFC) was the most significant factor in the interaction, and each element inside the loop was irreplaceable, which offered a potential opportunity for natural trypsin inhibitory drug design.

## Figures and Tables

**Table 1:** A summary of *K_i_* ^1^ values of Kunitzin-AH and its variants.

Name	Sequence	*K_i_* Value/µM	Hemolysis/µM
Kunitzin-AH	AVRPPFRCKAAFC	1.18 ± 0.08	>512
AH-1363	AVRPPFRCKAAF	96.94 ± 1.20 **	>512
AH-unoxidized	AVRPPFRCKAAFC	59.85 ± 2.82 **	>512
AH-1447	AVRPPFRCKAAKC	349.20 ± 0.93 ***	>512
AH-798	RCKAAFC	1.76 ± 0.13 *	>512
AH-837	RCKAAWC	14.74 ± 5.23 **	>512
AH-814	RCKAAYC	77.94 ± 7.84 **	>512
AH-880-I	RCKIIFC	16.05 ± 1.20 **	>512
AH-880-L	RCKLLFC	17.51 ± 1.20 *	>512
AH-884	RCKNNFC	133.20 ± 4.10 **	>512
AH-912	RCKQQFC	105.00 ± 5.02 **	>512
AH-770	RCKGGFC	169.20 ± 0.86 ***	>512

^1^*K_i_* value: inhibition constant of inhibitors, calculated by Morrison formula [26]. The smaller the constant is, the better the inhibitory activity. The smaller the constant is, the better the inhibitory activity. The formation of disulfide bonds between the Cys residues was underlined in the sequence row. The values were from three independent experiments with three technical replicates each. The statistical analysis was conducted between the *K_i_* values of the derivatives and Kunitzin-AH (* *p* < 0.05; ** *p* < 0.01; *** *p* < 0.001).

**Table 2:** Comparison of docking scores of selected sequences calculated by HDOCK.

Name	Sequence	Docking Scores ^1^
AH-798	RCKAAFC	−184.79
AH-837	RCKAAWC	−181.58
AH-884	RCKNNFC	−171.69

^1^ Docking score: a reflection of the affinity between two individual structures in the process of sampling. The values listed in the table were the highest-ranked scores among quantities of putative interaction modes [27].

## Data Availability

The mature peptide identified from the skin secretion was named Kunitzin-AH, and the nucleotide sequence of cDNA has been deposited in the Genbank database under an accession number: MZ231126.

## Supplementary Materials

The following are available online at https://www.mdpi.com/article/10.3390/pharmaceutics13070966/s1, Figure S1: Nucleotide and translated open-reading frame amino acid sequence of cloned cDNAs encoding a novel peptide precursor Kunitzin-AH from *Amolops hainanensis*. Putative signal peptides are marked with double-underline, mature peptides are marked with single-peptides and stop codons are indicated with asterisks, Figure S2: Identification of the specific trypsin inhibitor Kunitz-AH derived from the skin secretion of *Amolops hainanensis*. (a) RP-HPLC chromatogram of skin secretion of *A. hainanensis* monitored at 214 nm. The red arrow indicates the retention time of Kunitz-AH. (b) Annotated MS/MS spectrum of Kunitz-AH. (c) Predicted b- and y-ions arising from collision-induced dissociation of the double-charged (732.34 *m*/*z*, [M + 2H]^2+^) precursor ion. The observed b- and y-ions are indicated in blue and red typefaces; Figure S3: The progress curve of inhibition rate in the presence of different concentrations of Kunitzin-AH and its variants. Error bars represent the standard error of mean (S.E.M.) of three replicates, Figures S4 and S5, molecular dynamics simulation of the structures of AH-837 and AH-884. Table S1, Sequences and physicochemical properties of Kunitzin-AH and its derivatives.
