# Supplementary material for: Structure–Activity Relationship and Molecular Docking of a Kunitz-Like Trypsin Inhibitor, Kunitzin-AH, from the Skin Secretion of Amolops hainanensis
Source: Pharmaceutics. 2021 Jun 26;13(7):966. doi: 10.3390/pharmaceutics13070966 (PMC8309051; doi:10.3390/pharmaceutics13070966)
Supplement: Supplementary file 1 [file pharmaceutics-13-00966-s001.zip › pharmaceutics-1251864-SI.pdf]

pharmaceutics

MDPI

Supplementary Materials: Structure–Activity Relationship and Molecular Docking of a Kunitz-Like Trypsin Inhibitor, Kunitzin-AH, from the Skin Secretion of *Amolops hainanensis*

Yuqing Chen, Xinping Xi, Chengbang Ma, Mei Zhou, Xiaoling Chen, Zhuming Ye, Lilin Ge, Qinan Wu, Tianbao Chen, Lei Wang and Hang Fai Kwok

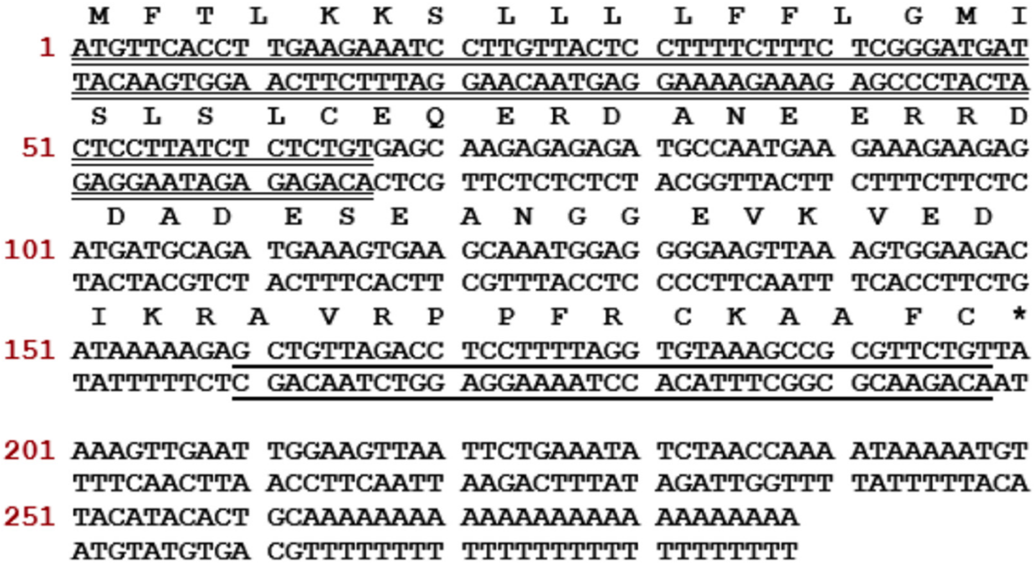

Figure S1. Nucleotide and translated open-reading frame amino acid sequence of cloned cDNAs encoding a novel peptide precursor Kunitzin-AH from Amolops hainanensis. Putative signal peptides are marked with double-underline, mature peptides are marked with single-peptides and stop codons are indicated with asterisks.

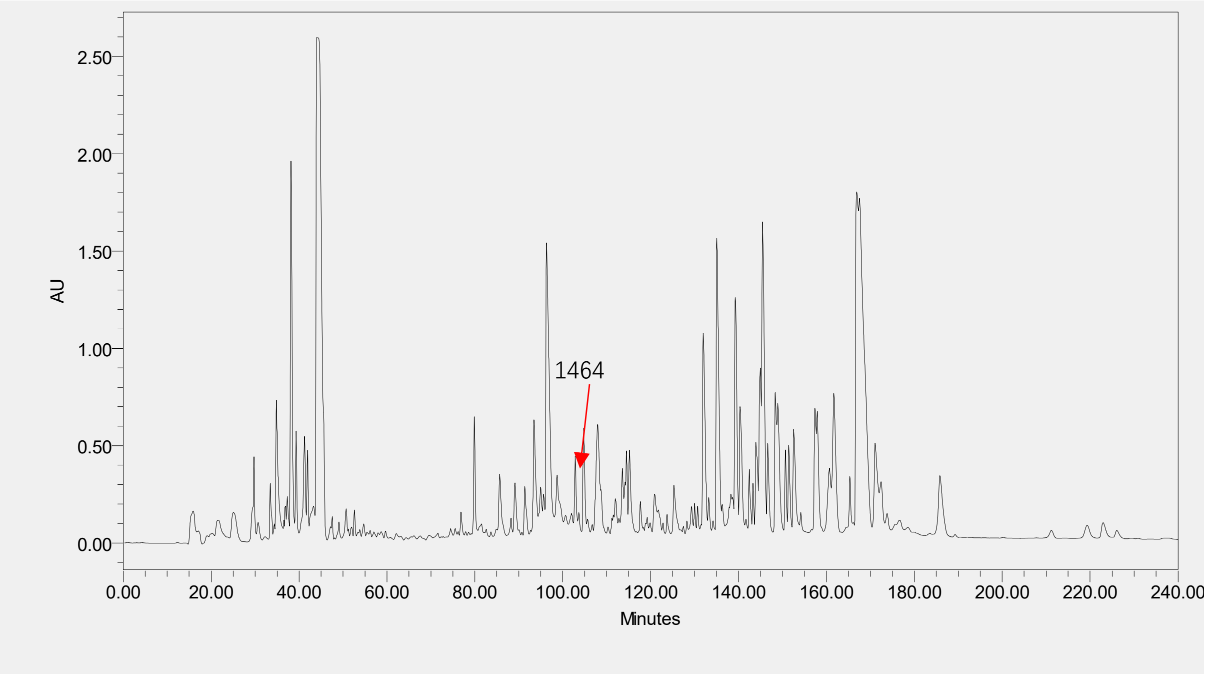

(a)

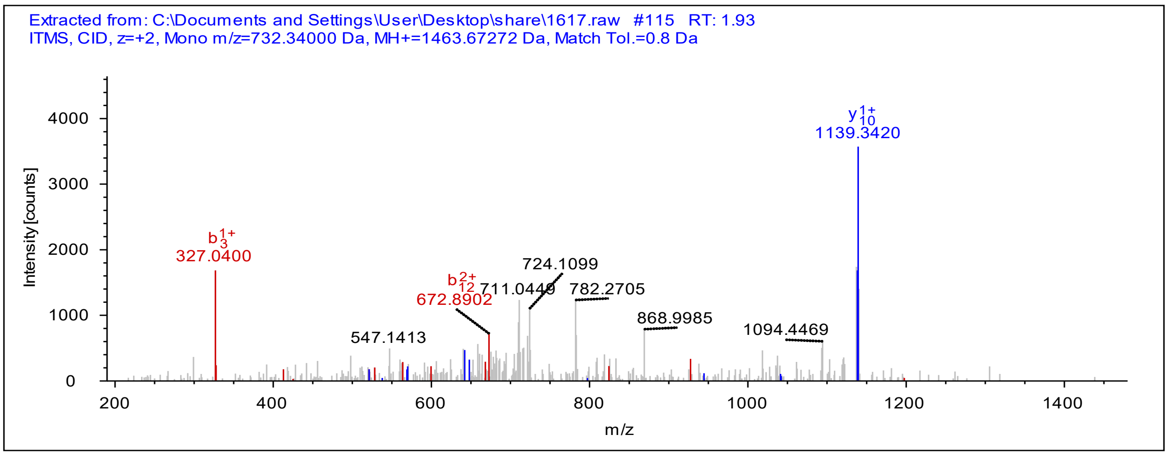

(b)

#1	b(1+)	b(2+)	Seq.	y(1+)	y(2+)	#2
1	72.04440	36.52584	A			13
2	171.11282	86.06005	V	1393.73446	697.37087	12
3	327.21394	164.11061	R	1294.66604	647.83666	11
4	424.26671	212.63699	P	1138.56492	569.78610	10
5	521.31948	261.16338	P	1041.51215	521.25971	9
6	668.38790	334.69759	F	944.45938	472.73333	8
7	824.48902	412.74815	R	797.39096	399.19912	7
8	927.49821	464.25274	C	641.28984	321.14856	6
9	1055.59318	528.30023	K	538.28065	269.64396	5
10	1126.63030	563.81879	A	410.18568	205.59648	4
11	1197.66742	599.33735	A	339.14856	170.07792	3
12	1344.73584	672.87156	F	268.11144	134.55936	2
13			C	121.04302	61.02515	1

(c)

Figure S2. Identification of the specific trypsin inhibitor Kunitz-AH derived from the skin secretion of Amolops hainanensis. (**a**) RP-HPLC chromatogram of skin secretion of A. hainanensis monitored at 214 nm. The red arrow indicates the retention time of Kunitz-AH. (**b**) Annotated MS/MS spectrum of Kunitz-AH. (**c**) Predicted b- and y-ions arising from collision induced dissociation of the double-charged (732.34 m/z, [M+2H]2+) precursor ion. The observed b- and y-ions are indicated in blue and red typefaces.

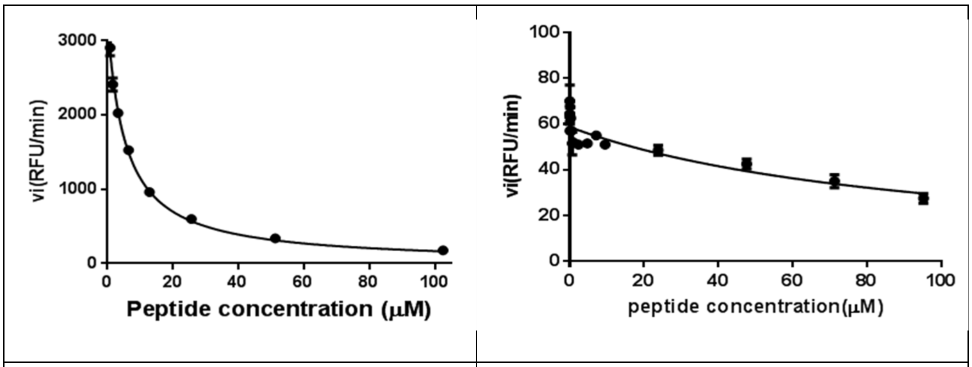

(a) Kunitzin-AH

(b) AH-1363

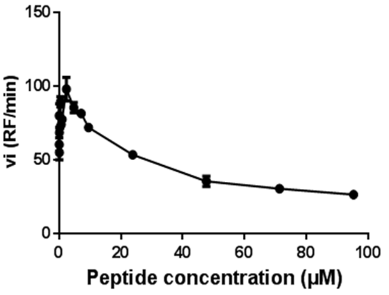

(c) AH-unoxidized

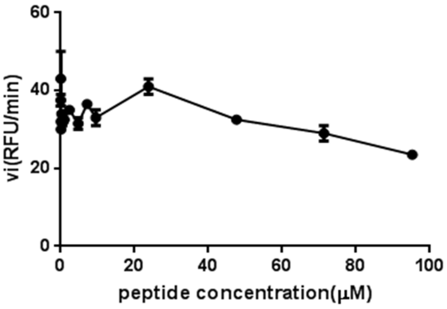

(d) AH-1447

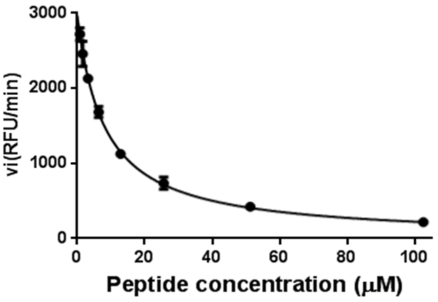

(e) AH-798

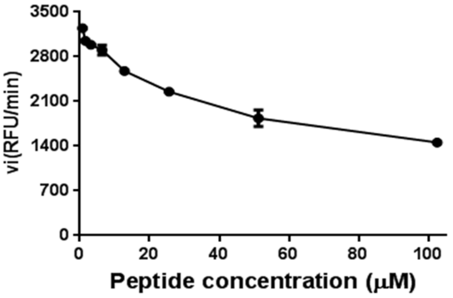

(f) AH-880-I

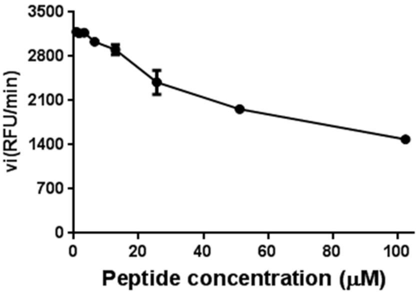

(g) AH-880-L

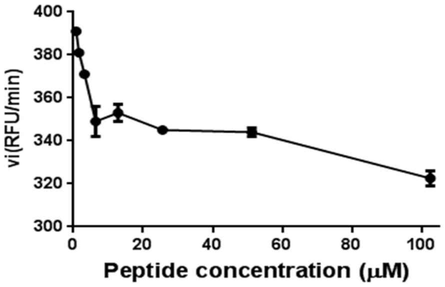

(h) AH-884

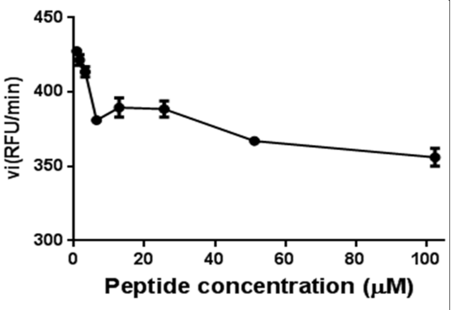

(i) AH-912

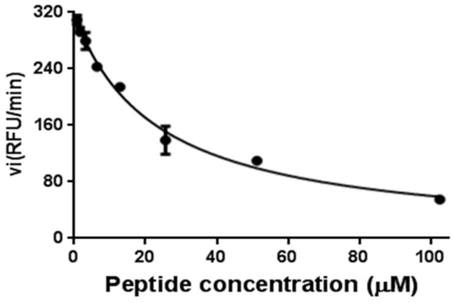

(j) AH-837

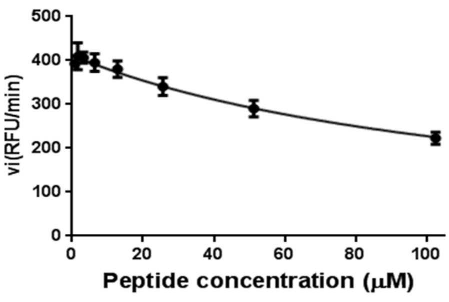	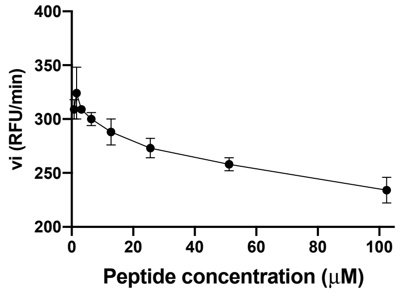
(k) AH-814	(l) AH-770

Figure S3. The progress curve of inhibition rate in the presence of different concentrations of peptides (**a**) Kunitzin-AH; (**b**) AH-1363; (**c**) AH-unoxidized; (**d**) AH-1447; (**e**) AH-798; (**f**) AH-880-I; (**g**) AH-880-L; (**h**) AH-884; (**i**) AH-912; (**j**) AH-837; (**k**) AH-814 and (**l**) AH-770 ranging from 0.8 μM to 102.4 μM. The Y-axis represented the rate of the fluorescence decay, while the X-axis represented peptide concentrations in μM. The Ki values were calculated by Morrison formula in Prism 6, in which Km = 11.70 μM, [S] = 42.86 μM, Et = 0.0002 μM. Error bars represent standard error of mean (SEM) of three replicates.

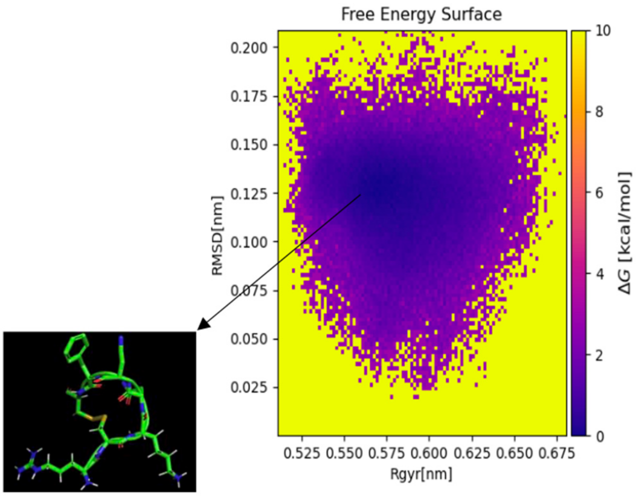

(a)

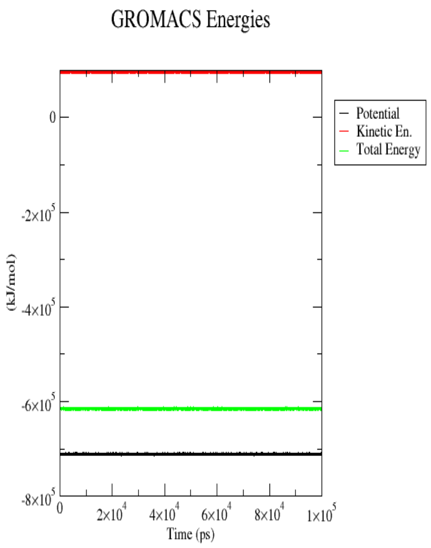

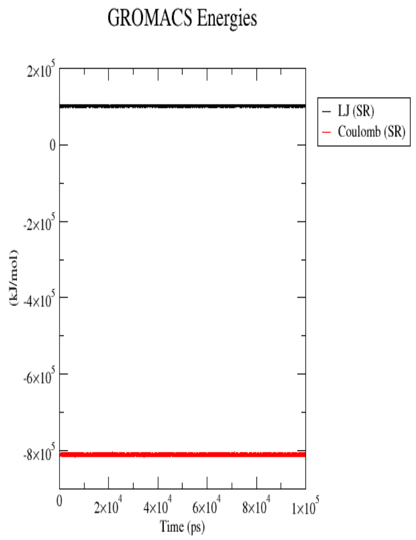

(b) **(c)**

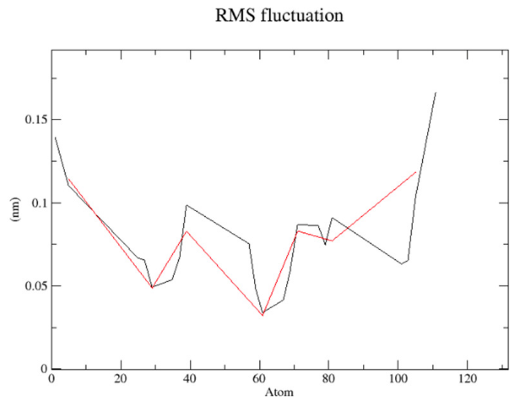

(d)

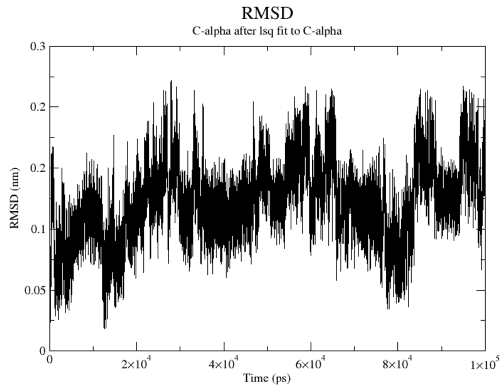

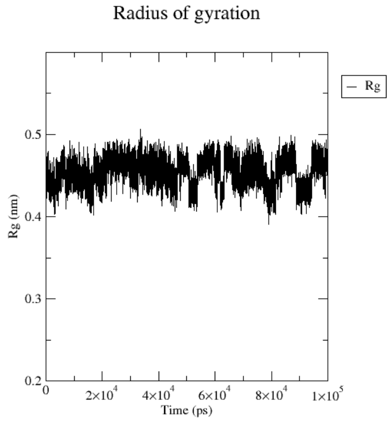

(e) **(f)**

Figure S4. (a) Free energy surfaces (in kcal/mol) for the aggregation of AH-837 forming a disulphide bridge in terms of the radius of gyration (Rgyr) and the root mean square deviation (RMSD). The darker region represented the lower energy of the structure, and the conformation with the lowest energy was indicated with an arrow. **(b)** Potential energy (black), kinetic energy

(red) and total energy (green) fluctuation of the system during the production molecular dynamics simulation within 100 ns; **(c)** Lennard-Jones Short Range potential (black) and Coulomb short-range force (red) fluctuation of the system during the production molecular dynamics simulation within 100 ns. **(d)** Root mean square fluctuation (RMSF) of the Cα in the structure of AH-837 (indicated with black lines) and the carbon backbone of the structure (indicated with red lines). **(e)** The root mean square deviation (RMSD) values of the Cα in the structure of AH-837 during the production molecular dynamics simulation within 100 ns; **(f)** Radius of gyration (Rg) of the whole conformation during the production molecular dynamics simulation within 100 ns.

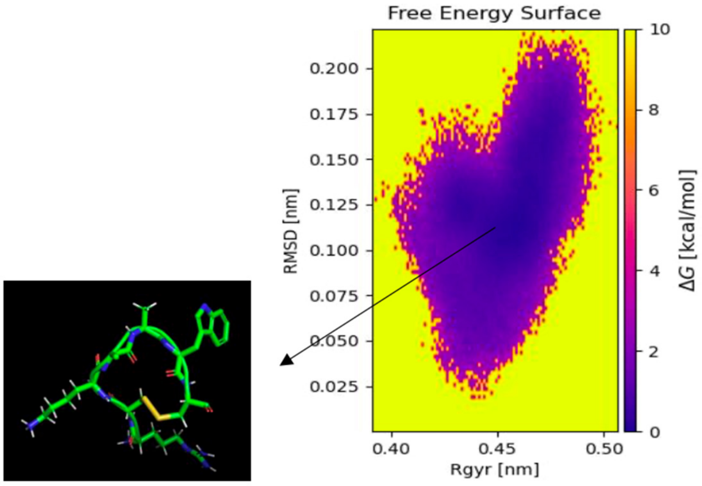

(a)

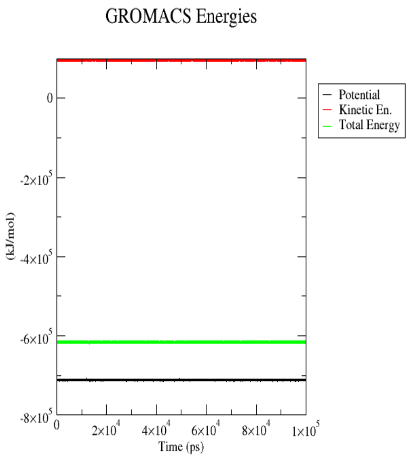

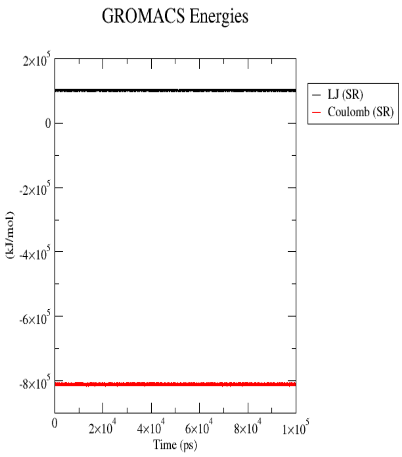

(b) **(c)**

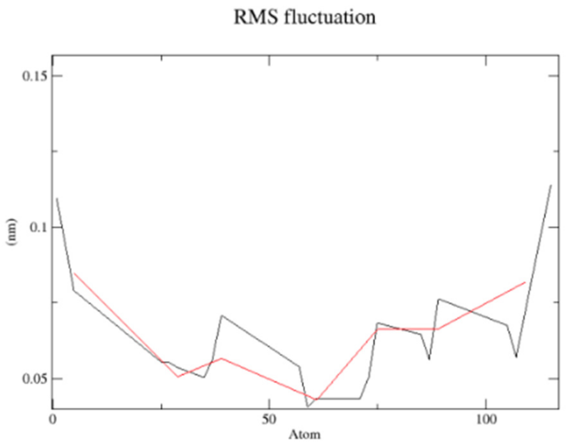

(d)

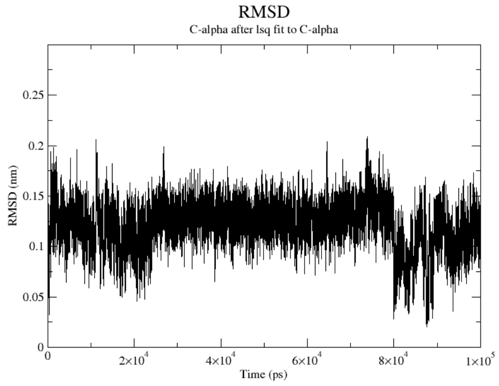

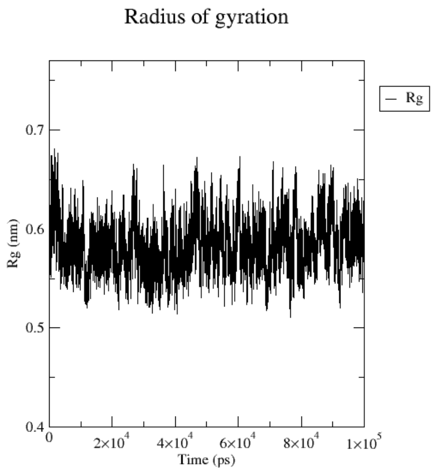

(e) **(f)**

Figure S5. (a) Free energy surfaces (in kcal/mol) for the aggregation of AH-884 forming a disulphide bridge in terms of the radius of gyration (Rgyr) and the root mean square deviation (RMSD). The darker region represented the lower energy of the structure, and the conformation with the lowest energy was indicated with an arrow. **(b)** Potential energy (black), kinetic energy (red) and total energy (green) fluctuation of the system during the production molecular dynamics simulation within 100 ns; **(c)** Lennard-Jones Short Range potential (black) and Coulomb short-range force (red) fluctuation of the system during the production molecular dynamics simulation within 100 ns. **(d)** Root mean square fluctuation (RMSF) of the Cα in the structure of AH-884 (indicated with black lines) and the carbon backbone of the structure (indicated with red lines). **(e)** The root mean square deviation (RMSD) values of the Cα in the structure of AH-884 during the production molecular dynamics simulation within 100 ns; **(f)** Radius of gyration (Rg) of the whole conformation during the production molecular dynamics simulation within 100 ns.
